# Differential Adaptation of *Propionibacterium freudenreichii* CIRM-BIA129 to Cow’s Milk *Versus* Soymilk Environments Modulates Its Stress Tolerance and Proteome

**DOI:** 10.3389/fmicb.2020.549027

**Published:** 2020-12-01

**Authors:** Florian Tarnaud, Floriane Gaucher, Fillipe Luiz Rosa do Carmo, Nassima Illikoud, Julien Jardin, Valérie Briard-Bion, Fanny Guyomarc’h, Valérie Gagnaire, Gwénaël Jan

**Affiliations:** ^1^INRAE, Institut Agro, STLO, Rennes, France; ^2^Bioprox, Levallois-Perret, France

**Keywords:** propionibacteria, soymilk, cow’s milk, proteomic, probiotic, stress

## Abstract

*Propionibacterium freudenreichii* is a beneficial bacterium that modulates the gut microbiota, motility and inflammation. It is traditionally consumed within various fermented dairy products. Changes to consumer habits in the context of food transition are, however, driving the demand for non-dairy fermented foods, resulting in a considerable development of plant-based fermented products that require greater scientific knowledge. Fermented soymilks, in particular, offer an alternative source of live probiotics. While the adaptation of lactic acid bacteria (LAB) to such vegetable substrates is well documented, little is known about that of propionibacteria. We therefore investigated the adaptation of *Propionibacterium freudenreichii* to soymilk by comparison to cow’s milk. *P. freudenreichii* grew in cow’s milk but not in soymilk, but it did grow in soymilk when co-cultured with the lactic acid bacterium *Lactobacillus plantarum*. When grown in soymilk ultrafiltrate (SUF, the aqueous phase of soymilk), *P. freudenreichii* cells appeared thinner and rectangular-shaped, while they were thicker and more rounded in cow’s milk utltrafiltrate (MUF, the aqueous phase of cow milk). The amount of extractable surface proteins (SlpA, SlpB, SlpD, SlpE) was furthermore reduced in SUF, when compared to MUF. This included the SlpB protein, previously shown to modulate adhesion and immunomodulation in *P. freudenreichii*. Tolerance toward an acid and toward a bile salts challenge were enhanced in SUF. By contrast, tolerance toward an oxidative and a thermal challenge were enhanced in MUF. A whole-cell proteomic approach further identified differential expression of 35 proteins involved in amino acid transport and metabolism (including amino acid dehydrogenase, amino acid transporter), 32 proteins involved in carbohydrate transport and metabolism (including glycosyltransferase, PTS), indicating metabolic adaptation to the substrate. The culture medium also modulated the amount of stress proteins involved in stress remediation: GroEL, OpuCA, CysK, DnaJ, GrpE, in line with the modulation of stress tolerance. Changing the fermented substrate may thus significantly affect the fermentative and probiotic properties of dairy propionibacteria. This needs to be considered when developing new fermented functional foods.

## Introduction

Fermented food products constitute a major source of live bacteria. In developed countries, the average consumption of fermented foods, and mainly fermented dairy products, supplies a daily dose of 10^10^ bacteria per person and per day (Rezac et al., 2018). This includes both LAB and propionibacteria. The latter are present at population levels close to 10^9^ CFU (Colony Forming Units) per gram in Swiss-type cheeses such as Emmental (Thierry et al., 1998). Propionibacteria are members of the *Propionibacteriaceae* family, which is divided into three genera: *Propionibacterium* spp., *Acidipropionibacterium* spp. and *Cutibacterium* spp. (Turgay et al., 2020). Among propionibacteria, the main species, *Propionibacterium freudenreichii*, which enjoys GRAS (Generally Recognized as Safe) and QPS (European Qualified Presumption of Safety) status, is widely acknowledged for its probiotic potential (Rabah et al., 2017). Probiotics are defined as live microorganisms which, when administered in adequate amounts, confer a beneficial health effect on the host (FAO/WHO, 2002). Because of their ability to modulate the gut microbiota and/or physiology, probiotic microorganisms are investigated for their potential role in the management of dysbiosis, antibiotic-associated diarrhea, *Helicobacter pylori* infection, necrotizing enterocolitis, traveler’s diarrhea, *Clostridium difficile* recurrence, ulcerative colitis, and irritable bowel syndrome (Sniffen et al., 2018). *Propionibacterium freudenreichii* is commercialized as a probiotic in functional food supplements in several countries including France (Sécuril, Yalacta), Finland (PJS, Valio) and Japan (BGF, Meiji).

*Propionibacterium freudenreichii* indeed constitutes a promising probiotic bacterium, although it has been less studied than LAB. In humans, its consumption modulates the gut microbiota in favor of bifidobacteria and at the expense of clostridia (Hojo et al., 2002; Seki et al., 2004). A modulation of human gut motility has also been reported following its consumption (Bouglé et al., 1999). One important probiotic potential is the ability of *P. freudenreichii* to mitigate inflammation in the context of colitis in animals (Foligné et al., 2010; Rabah et al., 2020). Its healing effects include the stimulation of Muc2 expression, limitation of gut permeability, and the protection of goblet cells and the general histological architecture of the intestinal mucosa (Plé et al., 2016; do Carmo et al., 2019; Ma et al., 2020). This ability, which is highly strain-dependent (Foligné et al., 2013), is mediated by key extractable surface proteins (Le Marechal et al., 2015; Deutsch et al., 2017; Ge et al., 2020). Indeed, mutational inactivation of the *slpB* gene was recently shown to affect *P. freudenreichii* surface properties, as well as its anti-inflammatory role, both *in vitro* and *in vivo* (do Carmo et al., 2017, 2018b, 2019). Accordingly, the SlpB protein purified from *P. freudenreichii* was shown to mitigate inflammation in human intestinal epithelial cells (do Carmo et al., 2017; Rabah et al., 2018a,b). Furthermore, *P. freudenreichii* was shown to produce extracellular vesicles containing the SlpB protein which are also involved in modulating host cell inflammation (Rodovalho et al., 2020). These results are in line with recent findings that identified propionibacteria as commensals within the gut microbiota of healthy infants (Colliou et al., 2017). The presence of commensal propionibacteria as a result of breast-feeding coincides with a lower incidence of necrotizing enterocolitis. A commensal strain isolated from an healthy infant was shown to mitigate intestinal inflammation via Th17 cell regulation (Colliou et al., 2017) and has also been shown to enhance neonatal host defenses against intestinal pathogen infection (Ge et al., 2019). Accordingly, a pilot clinical study reported the healing effect of freeze-dried *P. freudenreichii* cultures in the context of ulcerative colitis in humans (Suzuki et al., 2006; Mitsuyama et al., 2007). The consumption of an experimental cheese containing a selected immunomodulatory dairy strain of *P. freudenreichii* (isolated from cheese) as the only bacterium, reduced the intensity of colitis in mice (Plé et al., 2015). Furthermore, similar protection was observed using a cheese containing probiotic strains of both *P. freudenreichii* and *Lactobacillus delbrueckii* (Plé et al., 2016). Consuming such cheeses protected mice against trinitrobenzenesulfonic acid-induced colitis, alleviating symptom severity, and modulating local and systemic inflammation, as well as colonic oxidative stress and epithelial cell damage (Plé et al., 2016). These results suggest that it is possible to combine selected immunomodulatory dairy strains in order to develop an anti-inflammatory cheese. The beneficial metabolites produced by this bacterium include 1,4-dihydroxy-2-naphtoic acid (DHNA), a vitamin K2 (or menaquinone) biosynthesis intermediate, as well as 2-amino-3-carboxy-1,4-naphthoquinone (ACNQ) (Isawa et al., 2002; Furuichi et al., 2006). DHNA restores Lactobacilli and attenuates colitis in DSS-treated in mice (Okada, 2006). ACNQ enhances the activity of NADH peroxidase and NADH oxidase in Bifidobacteria (Mitsuyama et al., 2007). DHNA and ACNQ are considered to be the molecules responsible for the bifidogenic effect of propionibacteria metabolites (Hojo et al., 2002; Seki et al., 2004). In addition, the ability of *P. freudenreichii* to release vitamins B2 and B12 is exploited by the fermentation industry to produce food-grade vitamins, and it participates in increasing the nutritional value of food products fermented by this bacterium (LeBlanc et al., 2006; Thierry et al., 2011; Chamlagain et al., 2018; Assis et al., 2020). Finally, the production of short chain fatty acid propionate enables *P. freudenreichii* to modulate the proliferation/apoptosis balance both *in vitro* and *in vivo*, suggesting a role in preventing colon cancer (Cousin et al., 2012a, 2016).

Developed countries are seeing a shift in consumer habits from animal proteins to plant proteins. In recent years, the consumption of animal products has been questioned for several reasons: effects on health, environmental and ethical considerations, lifestyle changes, a demand for more dietetic foods, vegetarianism and veganism. Moreover, the current increase in the global population demands alternatives to animal proteins. There is therefore growing interest in plant alternatives to dairy products to fulfill this demand (Granato et al., 2010; Martins et al., 2013). Moreover, the increasing number of people who declare that they are lactose intolerant, the unfavorable cholesterol content of dairy products, allergies and vegetarianism all reinforce this interest in the development of non-dairy probiotic products such as fermented fruits and vegetables (Ranadheera et al., 2010; Peres et al., 2012; Martins et al., 2013). A wide variety of probiotic fermented non-dairy beverages is already produced worldwide using various matrices that include cereals, vegetables and legumes as the main raw material (Prado et al., 2008). Among these, legumes, and particularly soybean, are commonly used because of their agronomic value to soil nitrogen control and their nutritional richness. For example, soymilk contains high-quality proteins, isoflavones, unsaturated fatty acids and carbohydrates that are prebiotics and useful for the growth of bifidobacteria but can cause some digestive discomfort (Guillon and Champ, 2002). Soymilk is also cholesterol and lactose free (Scalabrini et al., 1998) so can be considered a good alternative to animal milk for the design of non-dairy probiotic fermented foods to meet the needs of specific populations (lactose intolerant and vegans). It can also provide protection against conditions such as cardiovascular disease, cancer and diabetes (Babashahi et al., 2015; Ewe and Yeo, 2015).

Retaining the benefits of fermented probiotic products involves the development of new fermented probiotic plant products. It has already been shown that soymilk is an adequate medium for the growth of bifidobacteria and various LAB such as *Streptococcus thermophilus*, *Lactobacillus acidophilus* and *Lactobacillus plantarum* (Wang et al., 2002, 2003). The use of LAB for the preparation of fermented soymilk has recently been the subject of increased attention (Santos et al., 2014; Harlé et al., 2020). However, little information is available in the literature regarding the behavior of *P. freudenreichii* in soymilk and other plant food matrices. In this paper, we investigated the suitability of soymilk as a substrate for growth and acidification by the probiotic strain *P. freudenreichii* CIRM-BIA129. We focused specifically on changes affecting proteome composition, cellular morphology and tolerance toward acid, bile salts and oxidative stresses when compared with cultivation in cow’s milk. This enabled us to further define the impact of the substrate (soymilk *vs.* cow’s milk), thereby providing an opportunity to explore the potential of *P. freudenreichii* to maximize the beneficial effects of soy products. In view of the physiological role of ingested bacteria, this work addressed the question of how dairy probiotic bacteria adapt to plant food matrices. Our findings open perspectives for the development of new functional fermented plant-based products.

## Materials and Methods

### Preparation of Cow’s Milk and Soymilk Ultrafiltrates

Cow’s milk ultrafiltrate (MUF) was prepared as previously described by our dairy platform at INRAE STLO (Michalski et al., 2006; Cousin et al., 2012b). Briefly, raw cow’s milk was skimmed prior to ultrafiltration using a UF pilot equipment equipped with a ceramic membrane, with a molecular weight cut-off point of 8 kDa. The overall composition of MUF was as follows: carbohydrate 5% (w/w); non-protein nitrogen 0.28% (w/w), minerals 0.75% (w/w) and dry matter 6.14% (w/w). MUF was supplemented with 5 g.L^–1^ food grade casein hydrolysate (Casein Peptone Plus, Organotechnie, La Courneuve, France), brought to pH 7 using NaOH, sterilized by 0.2 μm filtration (Nalgene, Roskilde, Denmark) and stored at 4°C.

A local company, Sojasun Technologies Triballat Noyal (Noyal-sur-Vilaine, France) supplied the soymilk ultrafiltrate (SUF) which was prepared according to patent N° EP 1 983 844 B1 (Efstathiou and Driss, 2010) and is commercially available under the designation BASOSOY (Triballat ingredients). Briefly, soybeans were dehulled prior to grinding in water, cooking under alkali conditions and eliminating the okara residue using a separator. The resulting soy juice was subjected to ultrafiltration to generate a retentate and a soy ultrafiltrate (SUF). The overall composition of the SUF was: carbohydrates 2.5% (w/w), protein 0.55% (w/w), non-protein nitrogen 0.20% (w/w), mineral 1% (w/w), dry matter 5% (w/w). The SUF was supplemented with 5 g.L^–1^ food grade soy hydrolysate (Bacto Soytone, BD Bioscience), brought to pH 7 using NaOH and autoclaved (110°C, 10 min). It was then centrifuged (28,000 × *g*, 30 min), filtered on Whatman paper (from 30 to 8 μm) and then on Nylon Net Filters (from 10 to 0.4 μm, Millipore), in order to remove insoluble compounds. It was finally sterilized by 0.2 μm filtration (Nalgene, Roskilde, Denmark) prior to storage at 4°C.

### Strains and Cultures

The *Propionibacterium freudenreichii* strain used in this study is stored and maintained by the CIRM-BIA International Biological Resource Center dedicated to bacteria of food interest (Centre International de Ressources Microbiennes-Bactéries d’Intérêt Alimentaire, INRAE, Rennes, France) with the number CIRM-BIA129. It was originally isolated and provided by CNIEL under the previous number ITG P20. *Lactobacillus plantarum* CIRM-BIA465 was provided by the CIRM-BIA. Propionibacteria were routinely precultured at 30°C without agitation and under microaerophilic conditions in Yeast Extract Lactate (YEL) medium as described by Malik et al. (1968). Lactobacilli were routinely precultured in De Man, Rogosa, Sharpe (MRS) medium at 30°C under the same conditions as described by De Man et al. (1960). For this study, two subcultures of the microbial strains were performed on the ultrafiltrate media described above, prior to their cultivation on cow’s milk or soymilk-based media.

In contrast to milk and soymilk, MUF and SUF are clear culture media that enable the monitoring of bacterial growth using spectrophotometry. In MUF and SUF, bacterial growth was followed by Optical Density (OD) (Spectrophotometer, DU 7400, Beckman, Fullerton, United States) at 650 nm and growth medium acidification using iCinac Wireless (AMS alliance, France) with an ISM probe (Mettler Toledo, France). As an alternative, *P. freudenreichii* was cultivated in UHT skimmed cow’s milk (Agrilait, Cesson-Sévigné, France), or in UHT soymilk (Sojasun Technologies Triballat Noyal) with or without the presence of *L. plantarum*. In cow’s milk and soymilk, propionibacteria and lactobacilli populations were determined by CFU counting (serial dilutions and plating). Propionibacteria were enumerated on YEL-agar after 6 days of anaerobic incubation at 30°C, while Lactobacilli were enumerated on MRS-agar after 2 days of anaerobic incubation at 30°C.

### Acetate and Propionate Quantification Using HPLC

After 48 h of culture, 1 ml of culture was sampled and centrifuged at 10,000 × *g* for 15 min at 4°C. The supernatant was filtered through a 0.22 μm sterile filter for further elimination of small particles, diluted (0.1 and 0.2) in H_2_SO_4_ 0.01N (0.005M) and then stored at −20°C. The thawed samples were further centrifuged (10,000 × *g*, 15 min, 4°C) to remove any precipitate then filtered through filters resistant to H_2_SO_4_ (Acrodisc LC 0.45 μm PVDF, Pall). The clear solution was collected for injection in the HPLC chromatograph. The concentrations of propionate and acetate were determined using a high-pressure liquid chromatography (HPLC) system (Dionex, Sunnyvale, CA, United States) equipped with an Aminex HPX-87H column (Biorad, Hercules, CA, United States), using 5 mM H_2_SO_4_ as the mobile phase as described by Garnier et al. (2020). Non-inoculated media were included as controls.

### Phase Contrast Microscopy, Atomic Force Microscopy and Transmission Electronic Microscopy

Propionibacteria were routinely examined as wet-mount fresh cultures using an immersion phase contrast × 100 objective on an Olympus BX51 optical microscope. As an alternative, cultures were dried on a mica slide prior to analysis using AFM (Atomic Force Microscopy, as previously described (Deutsch et al., 2012). Briefly, the bacteria were washed in HEPES-NaCl buffer, deposited onto a freshly cleaved disk of mica and allowed to dry for 24 h in a desiccator. AFM imaging was performed in air, at a controlled temperature of 20°C and using a MFP-3D-BIO microscope (Oxford Instruments, Asylum Research, Santa Barbara, CA, United States). Images were acquired in tapping mode using AC240TS cantilevers (Olympus, Tokyo, Japan). Transmission Electron Microscopy (TEM) was performed as described previously (Deutsch et al., 2010). Briefly, bacteria were washed in PBS, fixed using glutaraldehyde, postfixed using osmium tetroxide/potassium cyanoferrate/uranyl acetate and dehydrated in ethanol (30–100%) prior to embedding in Epon. Thin sections (70 nm) were collected on 200-mesh copper grids and counterstained with lead citrate before examination using a Philips CM12 transmission electron microscope.

### Stress Challenges Applied to Propionibacteria

Heat, oxidative, bile salt and acid challenges were applied to the cultures at the start of stationary phase (when the maximal OD was reached after 48 h of growth) as described previously (Leverrier et al., 2004; Gaucher et al., 2019a). The level of each challenge was fixed in order to trigger cell death in naïve propionibacteria but not in adapted ones. The heat challenge was performed by placing 2 mL (in a 15 mL polystyrene Falcon^TM^ tube) of *P. freudenreichii* culture in a water bath at 60°C for 10 min (Leverrier et al., 2004). The oxidative challenge was performed by adding 1.25 mM hydrogen peroxide (Labogros, France) to 2 mL *P. freudenreichii* culture for 1 h at 30°C (Serata et al., 2016; Gaucher et al., 2019a). For the acid challenge, cultures were centrifuged (6,000 × *g*, 10 min) and re-suspended in the same growth medium, but adjusted to pH 2.0 using HCl, prior to 1 h incubation at 30°C (Jan et al., 2001). The bile salts challenge was performed by adding 1 g.L^–1^ of a bile salts mixture (an equimolar mixture of cholate and deoxycholate; Sigma Chemical, St. Louis, MO, United States) to the culture before incubation for 1 h at 30°C (Leverrier et al., 2003). CFU counting was performed as described above, before and immediately after the challenge, in order to calculated percentage survival (Jan et al., 2001).

### Electrophoresis of Proteins

Whole-cell SDS protein extracts were prepared by disrupting bacterial pellets in SDS lysis buffer, prior to centrifugation to discard debris, as previously described (Jan et al., 2001). Surface extractible guanidine extracts were prepared as previously described (Le Marechal et al., 2015). Bacterial pellets were re-suspended in 5 M guanidine hydrochloride to reach a final OD_650_ of 20. After centrifugation (21,000 × *g*, 20 min, 30°C) to eliminate cells, the supernatant was dialyzed against 0.1% SDS in distilled water. The extracts were diluted in SDS sample buffer (Laemmli, 1970) prior to heat denaturation (10 min, 95°C). One-dimensional Polyacrylamide Gel Electrophoresis (12.5%) was conducted according to Laemmli’s procedure on a Protean II xi Cell (Bio-Rad, Hercules, United States) prior to Coomassie Blue-staining using Bio-Safe reagent (Bio-Rad).

### Label-Free Proteomics Analysis of Whole-Cell Protein Extracts

Label-free proteomics was performed as previously described (Gaucher et al., 2019a). Briefly, *P. freudenreichii* CIRM-BIA129 was cultivated in milk and soy ultrafiltrate until the start of stationary phase and 10 mL aliquots harvested by centrifugation (8,000 × *g*, 10 min, 20°C). The cells were washed twice with 10 mL PBS buffer (NaCl 8 g.L^–1^, KCl 2 g.L^–1^ KH_2_PO_4_ 2 g.L^–1^, Na_2_HPO_4_ 12H_2_O 35.8 g.L^–1^) and resuspended in 1 mL lysis solution [50 mM Tris-HCl [pH 7.5], 0.3% Sodium Dodecylsulfate (SDS), 200 mM dithiothreitol (DTT), 0.4 mM PMSF], and then sonicated immediately using a Vibra Cell sonicator (Bioblock Scientific, Illkirch, France). The cells were broken down using 0.1 mm zirconium beads (1 mL suspension of 10^10^ propionibacteria, 0.1 g of beads) in a Precellys Evolution homogenizer (Bertin Instruments, Montigny-le-Bretonneux, France). Cell debris were removed by centrifugation (20,000 × *g*, 10 min, 20°C) and the proteins extracts harvested. The proteins were further cleaned using the 2-D Clean-Up kit (GE Healthcare) and quantified with the 2-D Quant Kit. Tryptic digestion was performed on 100 μg whole-cell proteins from each sample for 15 h at 37°C using Sequencing Grade Modified Trypsin (Promega, Madison, United States) according to the manufacturer’s instructions and as described previously (Huang et al., 2018). Spectrophotometric-grade trifluoroacetic acid (TFA) (Sigma-Aldrich, United States) was added in order to stop tryptic digestion at pH 2.

Liquid chromatography and mass spectrometry were conducted as previously described (Huang et al., 2018). Briefly, experiments were performed using a nano RSLC Dionex U3000 system fitted to a Q-Exactive mass spectrometer (Thermo Scientific, San Jose, United States). The spectra of eluted peptides were recorded in full MS mode and selected within a mass range of 250–2000 m/z for MS spectra and a resolution of 70,000 at m/z 200. For each scan, the ten most intense ions were selected for fragmentation.

Protein identification was performed as previously described (Huang et al., 2018). Peptides were identified from the MS/MS spectra using X!Tandem pipeline software (Langella et al., 2017). The search was performed against the proteome of the *P. freudenreichii* CIRM-BIA129 strain (ITGP20) (downloaded from NCBI.nlm.nih.gov on 23 August 2018). For each peptide identified, a minimum score corresponding to an *e*-value below 0.05 was considered as a prerequisite for peptide validation, and a minimum of two peptides were required for protein identification.

Protein quantification was performed as previously described (Huang et al., 2018). Each peptide identified by tandem mass spectrometry was quantified using the free MassChroQ software before data treatment and statistical analysis under R software (R 3.2.2, Project for statistical computing). A specific R package called ‘MassChroqR’ was used to automatically filter dubious peptides for which the standard deviation of their retention time was longer than 30 s and to regroup peptide quantification data into proteins. For peak counting analysis, variance analysis was performed on proteins with a minimum peak ratio of 1.5 between the two culture conditions. Proteins with an adjusted *p*-value < 0.05 were considered to be significantly different.

For XIC based quantification, normalization was performed to take account of possible global quantitative variations between LC-MS runs. Peptides shared between different proteins were excluded automatically from the data set as well as peptides present in fewer than 85% of samples. Missing data were then imputed from a linear regression based on other peptide intensities for the same protein (Blein-Nicolas et al., 2015). Analysis of variance was used to determine proteins with significantly different abundances between our two culture conditions.

Proteins were considered to be differentially expressed if there was a significant (*p* < 0.05, ANOVA) change in expression of ≥2-fold (log2 ratio ≥ 1.5). A volcano plot was generated to visualize differentially expressed proteins in the core proteome of *P. freudenreichii* CIRM-BIA129 cultivated in SUF compared to MUF. Functional annotation and Clusters of Orthologous Groups (COGs) were obtained using the eggNOG-mapper v2 web tool (Huerta-Cepas et al., 2017, 2019).

### Statistical Analysis

The data were obtained from triplicate samples. All the results are presented as mean values with standard deviations. Statistical significance was set at *p* < 0.05. Calculations were performed using GraphPad Prism Software (Prism 7 for Windows).

## Results

### *Propionibacterium freudenreichii* and *Lactobacillus plantarum* Co-culture in Soymilk

**FIGURE 1:**
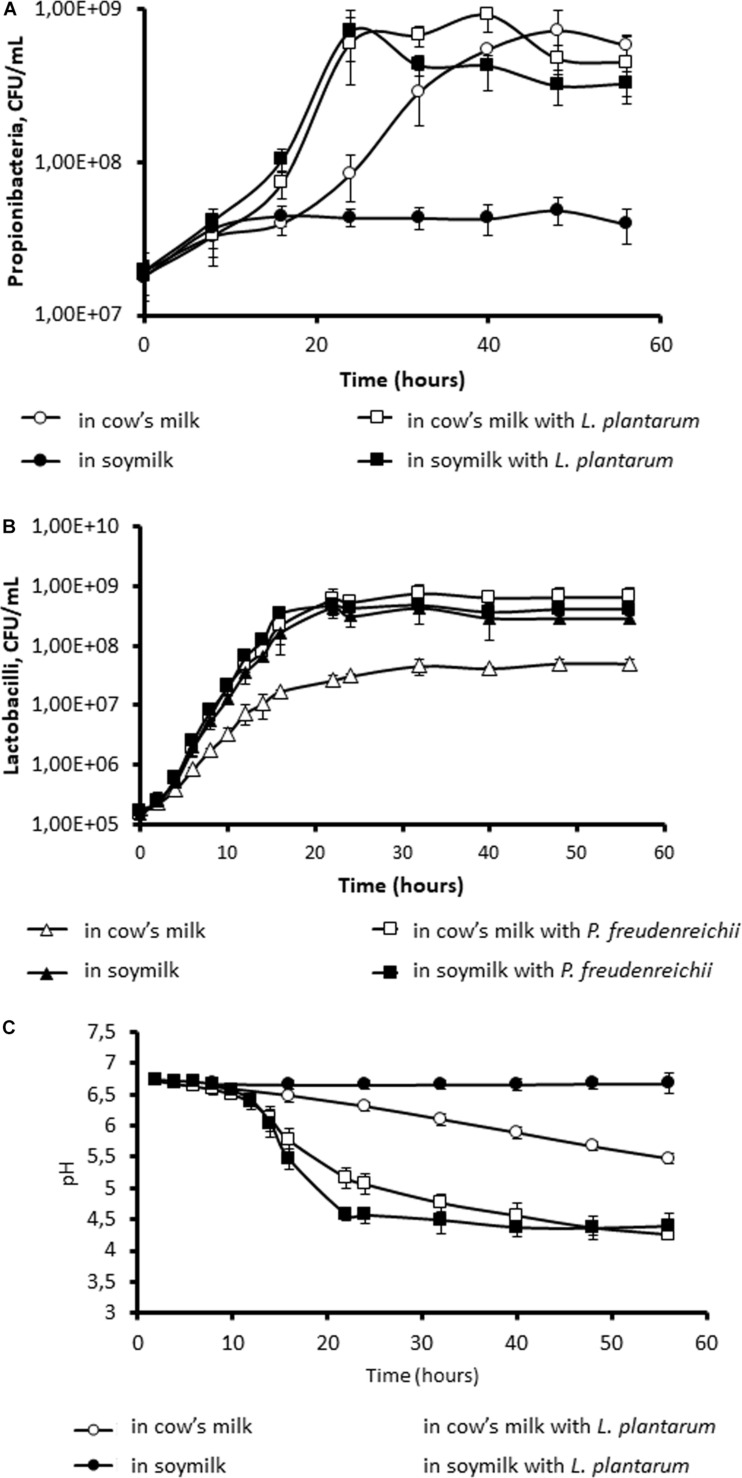
Co-culture with *Lactobacillus plantarum* allows the growth of *Propionibacterium freudenreichii* in soymilk. *P. freudenreichii* CIRM-BIA129 was cultivated in cow’s milk (○, □) or soymilk (●, ■), in pure culture (○, ●) or in co-culture with *L. plantarum* (■, □). Growth of propionibacteria **(A)** and of lactobacilli **(B)** was monitored by CFU counting. Acidification was monitored **(C)**.

We first of all investigated the ability of *P. freudenreichii* to grow in either UHT cow’s milk or UHT soymilk. As shown in Figure 1, *P. freudenreichii* grew in UHT cow’s milk to reach a final population of 6 × 10^8^ CFU.mL^–1^. Interestingly, this growth was facilitated by the co-culture with *L. plantarum*. *P. freudenreichii* CIRM-BIA129 is indeed known to metabolize lactose (Falentin et al., 2016), as well as the lactate produced by LAB (Langsrud and Reinbold, 1973; Thierry et al., 1998). The final population was similar, but growth started earlier. By contrast, in soymilk, the growth of *P. freudenreichii* alone was extremely limited, so that the final population did not exceed 4 × 10^7^ CFU.mL^–1^. However, it reached a final population of 4 × 10^8^ CFU.mL^–1^ in co-culture with *L. plantarum*. The presence of the lactic acid bacterium thus enabled growth of the Propionibacterium in soymilk (Figure 1A). The population of Lactobacilli reached 7.2 10^8^ CFU/mL, while that of Propionibacteria reached 3.3 10^8^ CFU.mL^–1^. Accordingly, the pH of soymilk remained constant during the monoculture of *P. freudenreichii* in soymilk, while acidification was observed in cow’s milk (Figure 1B). Moreover, co-culture with *L. plantarum* resulted in more pronounced acidification, down to 4.25 in cow’s milk and 4.39 in soymilk. This evidenced collaboration between *P. freudenreichii* and *L. plantarum* in terms of growth and metabolism in soymilk. As an initial hypothesis, non-protein nitrogen may constitute the limiting factor for the growth of non-proteolytic *P. freudenreichii*, while *L. plantarum* would supply the necessary non-protein nitrogen because of its potential proteolytic ability. As a second hypothesis, *L. plantarum* may supply propionibacteria with lactic acid, their preferred carbon source. *P. freudenreichii* was unable to metabolize sucrose and raffinose, which are the main carbohydrates present in soymilk, as evidenced by API gallery (results not shown), but only glucose and galactose that are also present in soymilk.

### *Propionibacterium freudenreichii* Requirement for Non-protein Nitrogen in Soymilk Ultrafiltrate

**FIGURE 2:**
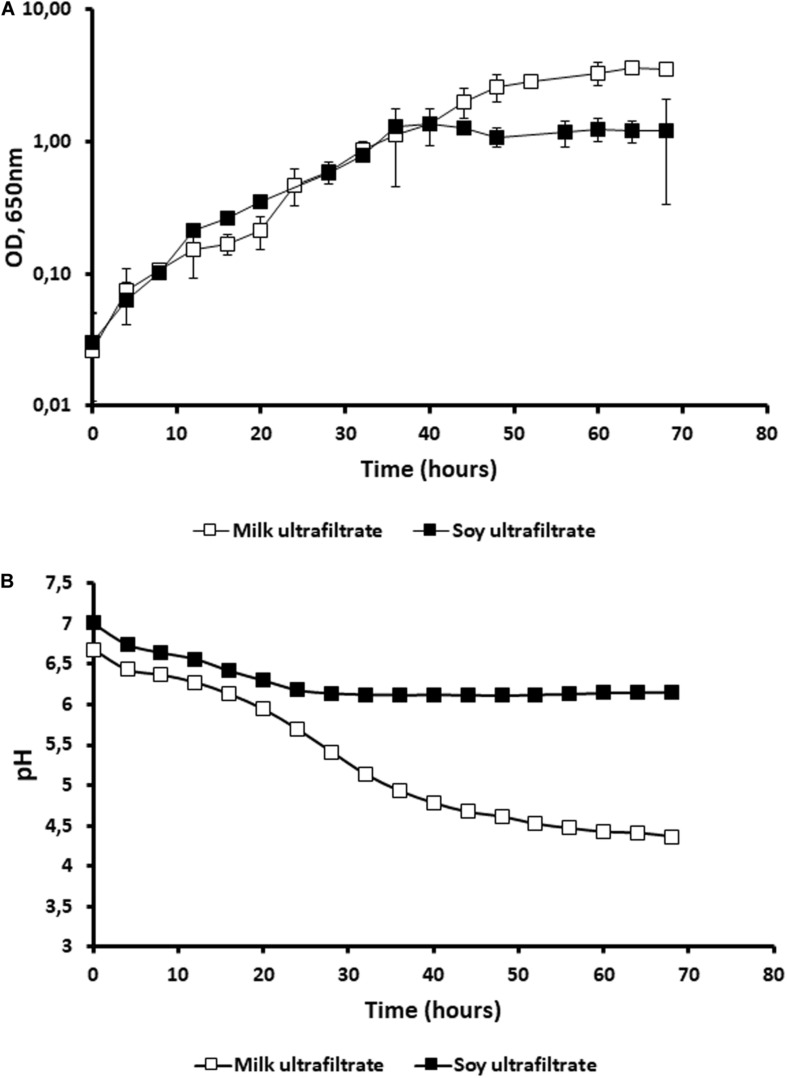
The addition of peptone allows the growth of *Propionibacterium freudenreichii* in cow’s milk ultrafiltrate (MUF) and in soymilk ultrafiltrate (SUF). *P. freudenreichii* CIRM-BIA129 was cultivated in MUF [cow’s milk ultrafiltrate supplemented with casein peptone (□)], or in SUF [soymilk ultrafiltrate supplemented with soy peptone (■)], in a pure culture. The growth of propionibacteria was monitored by following turbidity (OD at 650 nm) during incubation at 30°C **(A)** and acidification was also monitored **(B)**.

When we used ultrafiltrate of cow’s milk (MUF) and ultrafiltrate of soymilk (SUF), no growth of *P. freudenreichii* was observed in either ultrafiltrate (data not shown). Indeed, the nitrogen content of these media is low, although carbohydrates are present and not limiting (see section “Materials and Methods”). By contrast, the addition of soy peptone to SUF and that of casein peptone to MUF both led to an increase in the OD (Figure 2A). Accordingly, no acidification was observed in the raw ultrafiltrates in the absence of peptone. The addition of soy peptone to SUF caused a slight drop of pH from 7 to 6.5, in agreement with the small quantities of glucose and galactose available (Figure 2B). The addition of casein peptone to MUF enabled the growth of *P. freudenreichii*, with acidification down to 4.5 because of the lactose content, which was not limiting, i.e., 49 g.L^–1^ (Figure 2B). The control media MUF and SUF did not contain acetate and propionate. After fermentation by *P. freudenreichii*, the acetate concentration was 0.63 g.L^–1^ and the propionate 0.83 g.L^–1^ in SUF. The values of both organic acids were higher in the MUF: 1.23 g.L^–1^ acetate, 3.42 g.L^–1^ propionate.

### Different Morphologies of *Propionibacterium freudenreichii* in Cow’s Milk Versus Soymilk Environments

**FIGURE 3:**
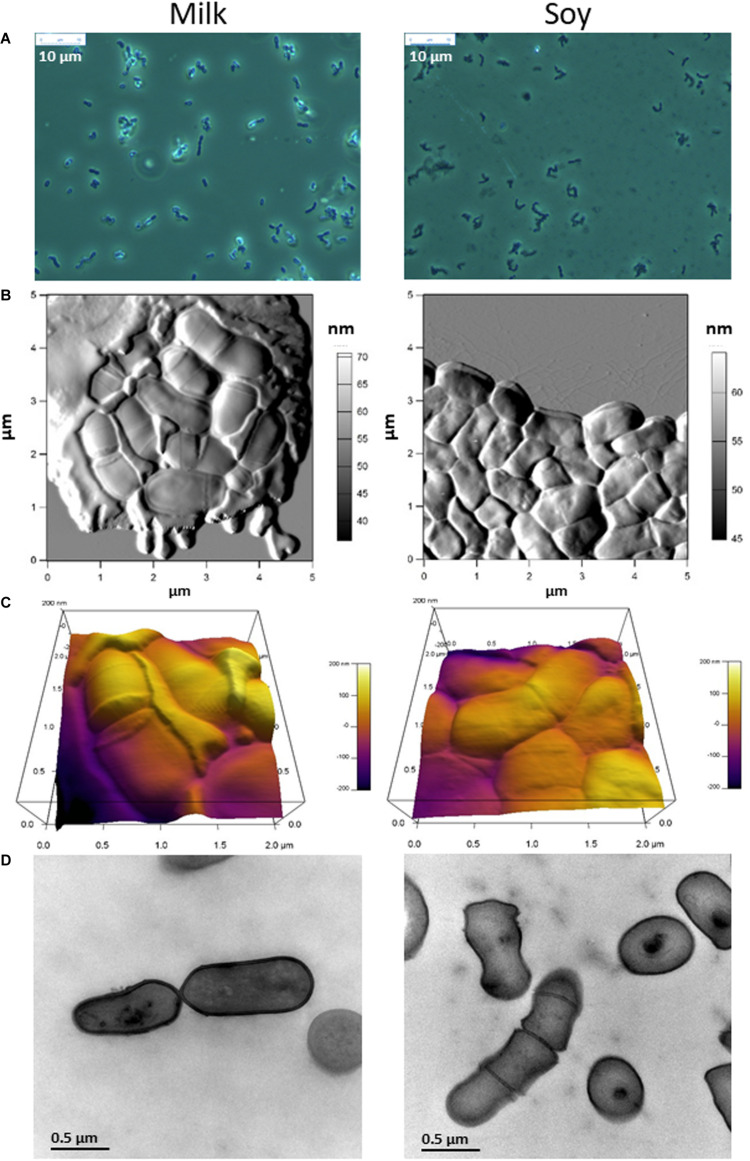
The morphology of *Propionibacterium freudenreichii* is different during growth in cow’s milk ultrafiltrate (MUF) and soymilk ultrafiltrate (SUF). *P. freudenreichii* CIRM-BIA129 was cultivated in MUF supplemented with casein peptone (left) or in SUF supplemented with soy peptone (right), in a pure culture. Cultures were observed using phase contrast photon microscopy **(A)**, atomic force microscopy three dimensional amplitude **(B)** and height **(C)** or transmission electron microscopy **(D)**.

We examined the morphology of *P. freudenreichii*, comparing the changes observed after growth in MUF with added casein peptone, and in SUF with added soy peptone, at three different microscopic scales. First, fresh-mount phase contrast examination revealed a refringent and shiny aspect around propionibacteria cultivated in MUF, but not in SUF (Figure 3A). Second, propionibacteria appeared to be surrounded by an extracellular compound in cow’s milk but not in soy ultrafiltrate, when examined using AFM (Figure 3B). By zooming and using a 3D height view, propionibacteria appeared to be embedded within an extracellular matrix when grown in MUF, but not in SUF (Figure 3C). Bacteria grown in MUF maintained a rod-like shape with round edges, while those grown in SUF seemed to deform when closely packed, as indicated by the sharp boundaries between neighbors. The length and width of *P. freudenreichii* individuals were respectively 1.13 ± 0.26 and 0.59 ± 0.10 μm in MUF *vs.* 1.11 ± 0.19 and 0.65 ± 0.08 μm in SUF (*p* > 0.1; *N* = 14), indicating that their dimensions were not totally dependent on the culture medium. However, the thickness of individual bacteria grown in SUF and then dried was only 0.21 ± 0.03 μm (*N* = 7), *vs.* 0.41 ± 0.04 μm (*N* = 13) when grown in cow’s MUF and then dried (*p* < 0.0001). Finally, transmission electron microscopic examination of bacterial sections further confirmed major differences in morphology, with round-shaped propionibacteria in MUF with a well-defined cell wall surrounding the bacteria, but rectangular-shaped ones in SUF with less clearly defined cell wall limits.

### Different Stress Tolerance of *Propionibacterium freudenreichii* in Cow’s Milk Versus Soymilk Environments

**FIGURE 4:**
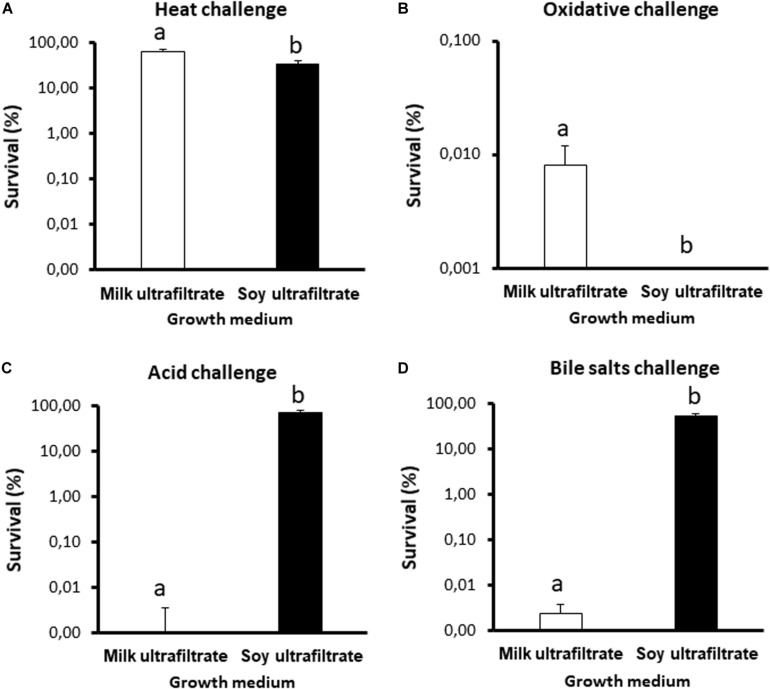
The stress tolerance of *Propionibacterium freudenreichii* is different during growth in cow’s milk ultrafiltrate (MUF) or soymilk ultrafiltrate (SUF). *P. freudenreichii* CIRM-BIA129 was cultivated in MUF or SUF. The cultures were then subjected to a heat challenge (**A**, 55°C for 30 min), oxidative challenge (**B**, 1.15 mM H_2_O_2_ for 1 h), acid challenge (**C**, pH = 2 for 1 h), and bile salts challenge (**D**, 1 gL^–1^ for 1 h), as described in section “Materials and Methods.” *P. freudenreichii* viability was determined by CFU counting before and after the challenges. The results are expressed as percentage survival. Error bars represent the standard deviations for triplicate experiments. Significant differences are reported using different letters above the columns (*p* ≤ 0.05).

In view of the major differences observed, we further investigated stress tolerance under both conditions, focusing on challenges relevant to the processing and digestion of probiotics (Figure 4). Propionibacteria were significantly more sensitive to heat and oxidative challenges when grown in SUF (Figures 4A,B), but were also significantly more tolerant toward acid and bile salts challenges (Figures 4C,D). Such major modulations of stress tolerance suggest that the fermented substrate also modulated the expression of key stress adaptation proteins.

### Different Proteomes of *Propionibacterium freudenreichii* in Cow’s Milk Versus Soymilk Environments

**FIGURE 5:**
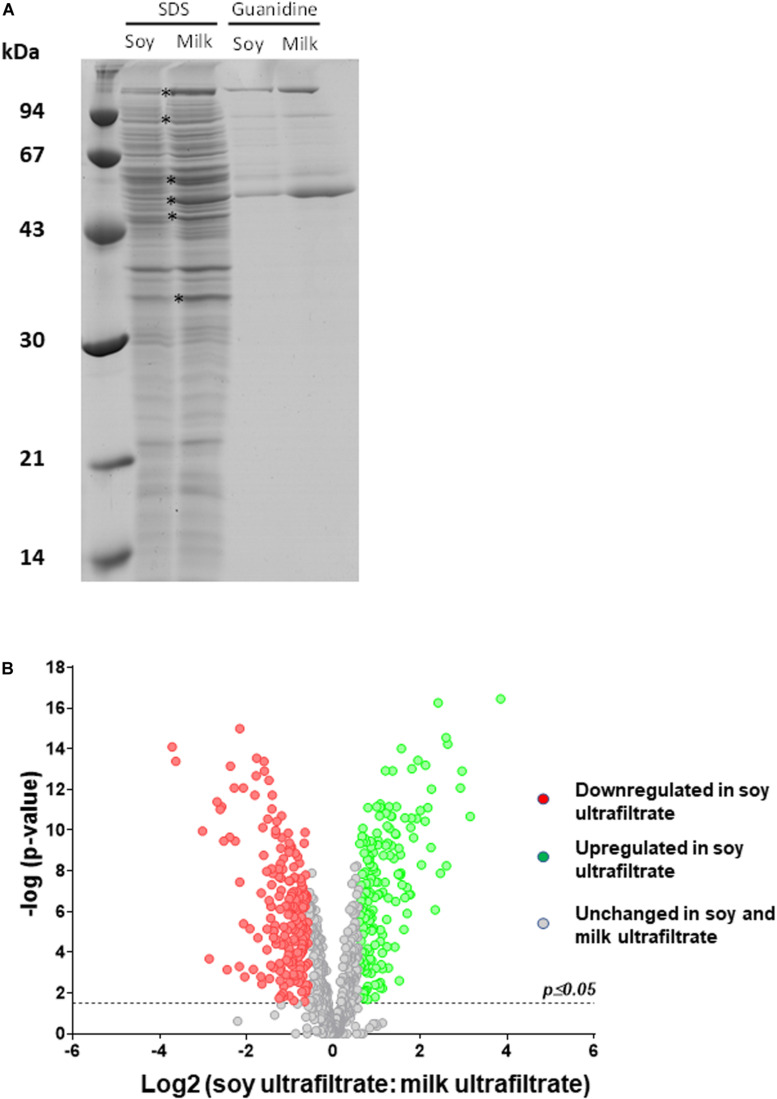
The cellular proteome of *P. freudenreichii* is different during growth in cow’s milk ultrafiltrate (MUF) and soymilk ultrafiltrate (SUF). *P. freudenreichii* CIRM-BIA129 was cultivated in MUF or SUF. **(A)** Whole-cell SDS extracts and surface guanidine extracts were separated using SDS-PAGE. The proteins which constitute the surface extractible layer are indicated. **(B)** Global cellular proteomes were analyzed by label-free proteomics using trypsinolysis and nano-LC-MS/MS. The Volcano plot shows a Log(2) fold change of the differentially expressed proteins of *Propionibacterium freudenreichii* CIRM BIA129 strain cultivated in SUF vs. MUF. Green (up-regulated proteins) and red (down-regulated proteins) circles indicate proteins that were statistically different (*p* ≤ 0.05) in terms of their abundance in soymilk and cow’s milk by twofold or more.

We therefore investigated how the *P. freudenreichii* proteome varied, comparing cow’s milk and soy ultrafiltrates as growth media. As shown in Figure 5, SDS-PAGE analysis of SDS whole-cell extracts suggested quantitative and qualitative differences in the cellular proteome. In particular, a set of proteins (indicated by asterisks) appeared to be more strongly expressed when propionibacteria were grown on MUF. In line with this, an analysis of guanidine extracts confirmed differences in terms of surface extractable proteins. In particular, a protein band previously attributed to InlA (145 kDa), as well as another band which might include SlpA and/or SlpB (57–58 kDa), appeared to be more abundant when propionibacteria were grown on MUF.

In order to obtain further insight into variations of the proteome, whole-cell protein extracts were then analyzed by label free proteomics using nano-LC-MS/MS. All proteins with significantly different levels as a function of their growth medium are listed in Supplementary Table S1. Table 1 presents proteins that were differentially expressed among those previously shown to be surface-exposed (Le Marechal et al., 2015). This analysis agreed well with the electrophoretic analysis (Figure 5A) and revealed differences in surface proteins. Indeed, InlA, SlpB and SlpE were more abundant in MUF, while SlpA was more abundant in SUF.

**TABLE 1:** Surface extractable proteins modulated during *P. freudenreichii* growth in soy ultrafiltrate compared to cow’s milk ultrafiltrate.

Accession	Description^(a)^	Ratio^(b)^	Adjusted *p*-value
**CDP47912.1**	Resuscitation-promoting factor RpfB	2.34	2.5E-06
**CDP49065.1**	Surface layer protein A	1.56	3.1E-07
**CDP47874.1**	60 kDa chaperonin 1 (Protein Cpn60 1) (groEL protein 1) (Heat shock protein 60 1)	0.66	2.2E-07
**CDP49357.1**	Hypothetical protein PFCIRM129_11455	0.60	8.7E-03
**CDP49418.1**	Surface layer protein D	0.45	1.5E-02
**CDP49444.1**	Secreted transglycosydase	0.30	2.0E-05
**CDP48888.1**	Iron transport system substrate-binding protein ABC transporter	0.21	8.4E-13
**CDP48273.1**	Surface layer protein B	0.17	7.1E-12
**CDP48858.1**	Surface layer protein E	0.16	4.1E-12

*^(*a*)^Determined using a database composed of the proteome of *P. freudenreichii* CIRM-BIA129 (downloaded from NCBI.nlm.nih.gov on 23 August 2018). ^(b)^Ratio between protein levels in the soy ultrafiltrate versus the cow’s milk ultrafiltrate. This ratio indicates induction (ratio > 1.5) or repression (ratio < 0.66) during growth in soy ultrafiltrate.*

**FIGURE 6:**
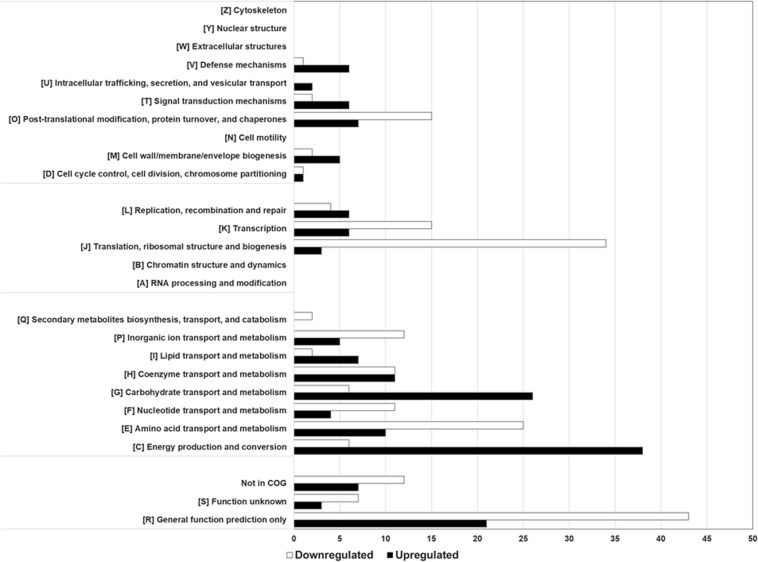
Breakdown of differential proteins in biological processes. The functional distribution and biological processes were predicted based on the functional classifications of the COG database. Comparing *P. freudenreichii* CIRM-BIA129 cultivated in SUF versus MUF, proteins upregulated in SUF are presented in black, proteins downregulated in SUF in white.

A volcano plot was generated to visualize the amount of differentially expressed proteins (*p* < 0.05) in the soy *vs.* cow’s milk media (Figure 5B). A total of 812 proteins (32% of the predicted proteome, according to the genome sequence) were identified and differences in protein abundance were deduced from proteomic quantitative analysis. More precisely, 175 up-regulated and 199 down-regulated proteins were detected when *P. freudenreichii* was cultivated on a soy base, while 438 proteins remained unaffected. The 374 affected proteins were classified and belonged to various COG functional categories (Figure 6) (Tatusov, 2000). Within the “metabolism” categories (C, E, F, G), many proteins were upregulated in soy. This notably included proteins involved in carbohydrate transport and metabolism (26 proteins upregulated, Table 2), as well as energy production and conversion (38 proteins upregulated, Table 3). By contrast, proteins involved in “amino acid transport and metabolism” (26 proteins) were downregulated (Table 4). Furthermore, proteins classified in the “translation, ribosomal structure and biogenesis” category were also downregulated (34 proteins). It is noteworthy that the “Post-translational modification, protein turnover, and chaperones” category includes a set of proteins that are downregulated, including groEL 1 and GroEL2, DnaJ2, HSP-70 cofactor 1 and the S-layer proteins SlpB and SlpE.

**TABLE 2:** Proteins in the G category (Carbohydrate transport and metabolism) modulated during *P. freudenreichii* growth in soy ultrafiltrate compared to cow’s milk ultrafiltrate.

Accession	Description^(a)^	Ratio^(b)^	Adjusted *p*-value
**CDP49431.1**	Glycosyltransferase (glycogen synthase)	4.77	9.6E-13
**CDP49219.1**	PTS enzyme I	4.31	3.7E-11
**CDP48882.1**	Phosphoglycerate mutase/Fructose-2,6-bisphosphatase	3.46	2.6E-11
**CDP49180.1**	iolC (Myo-inositol catabolism iolC protein)	3.34	1.5E-07
**CDP47782.1**	Hypothetical protein PFCIRM129_07365	2.92	1.3E-08
**CDP49320.1**	Glucose-6-phosphate 1-dehydrogenase	2.90	1.6E-08
**CDP48739.1**	Phosphoketolase pyrophosphate	2.68	1.5E-10
**CDP48828.1**	Ribose-5-phosphate isomerase 2	2.62	2.5E-09
**CDP48788.1**	Phosphoglucomutase	2.56	1.2E-13
**CDP49543.1**	Glycerate kinase GlxK/GarK	2.50	2.0E-11
**CDP48996.1**	Galactokinase	2.42	7.1E-12
**CDP48782.1**	Glycosyl hydrolase, family 13 (putative alpha-amylase, catalytic domain)	2.35	5.4E-05
**CDP48834.1**	Transketolase	2.08	2.0E-11
**CDP49788.1**	Galactokinase	2.03	3.7E-08
**CDP48837.1**	Aldose 1-epimerase	1.95	3.5E-08
**CDP49042.1**	UTP–glucose-1-phosphate uridylyltransferase	1.87	8.5E-07
**CDP49226.1**	Glucose-6-phosphate isomerase (GPI) (Phosphoglucose isomerase) (PGI) (Phosphohexose isomerase) (PHI)	1.82	6.7E-10
**CDP49568.1**	Polyphosphate glucokinase	1.80	1.3E-06
**CDP49821.1**	Histidine triad (HIT) protein	1.70	7.4E-03
**CDP48650.1**	Phosphoglycerate mutase (D-phosphoglycerate 2,3-phosphomutase)	1.68	4.2E-08
**CDP48024.1**	Transaldolase 1	1.68	4.0E-10
**CDP49678.1**	Pyruvate phosphate dikinase	1.63	1.3E-09
**CDP47731.1**	DhaK PTS-dependent dihydroxyacetone kinase, dihydroxyacetone-binding subunit	1.59	2.0E-07
**CDP49834.1**	Ribose-5-phosphate isomerase 3	1.58	9.8E-05
**CDP49177.1**	iolG1 (Myo-inositol catabolism IolG1 protein) (myo-inositol 2-dehydrogenase)	1.56	1.9E-07
**CDP49639.1**	Gluconate kinase (Gluconokinase)	1.51	2.5E-04
**CDP49220.1**	Phosphocarrier, HPr family	0.57	1.3E-03
**CDP48825.1**	Triosephosphate isomerase 2	0.55	2.6E-07
**CDP47629.1**	Glycogen debranching enzyme GlgX	0.51	1.7E-07
**CDP49749.1**	Phosphoglycerate mutase/Fructose-2,6-bisphosphatase	0.49	2.4E-07
**CDP48965.1**	Nucleoside-diphosphate-sugar epimerases	0.43	7.8E-10
**CDP49513.1**	Alpha-1,4-glucosidase	0.28	7.0E-04

*^(a)^Determined using a database composed of the proteome of *P. freudenreichii* CIRM-BIA129 (downloaded from NCBI.nlm.nih.gov on 23 August 2018). ^(b)^Ratio between the protein level in soy ultrafiltrate and in cow’s milk ultrafiltrate. This ratio indicates induction (ratio > 1.5) or repression (ratio < 0.66) during growth in soy ultrafiltrate.*

**TABLE 3:** Proteins in the C category (Energy production and conversion) modulated during *P. freudenreichii* growth in soy ultrafiltrate compared to cow’s milk ultrafiltrate.

Accession	Description^(a)^	Ratio^(b)^	Adjusted *p*-value
**CDP48125.1**	FAD-dependent pyridine nucleotide-disulphide oxidoreductase:4Fe–4S ferredoxin, iron–sulfur binding:Aromatic-ring hydroxylase	7.77	1.3E-13
**CDP49141.1**	Oxidoreductase	6.18	6.0E-15
**CDP48084.1**	FAD linked oxidase domain protein	6.03	2.8E-15
**CDP49140.1**	Iron-sulfur protein	5.31	5.5E-17
**CDP48450.1**	Succinate dehydrogenase, subunit A	5.06	8.3E-07
**CDP48130.1**	Glycerol-3-phosphate dehydrogenase	3.85	3.8E-14
**CDP49203.1**	Lactaldehyde dehydrogenase	3.77	2.7E-11
**CDP48743.1**	Anaerobic glycerol-3-phosphate dehydrogenase subunit B	3.49	9.7E-14
**CDP48004.1**	NADH-quinone oxidoreductase chain D (NADH dehydrogenase I, chain D)	3.24	1.2E-06
**CDP48124.1**	Pyruvate synthase/Pyruvate-flavodoxin oxidoreductase	3.23	6.6E-08
**CDP49123.1**	Aldo/keto reductase	3.12	2.1E-07
**CDP48449.1**	Succinate dehydrogenase, subunit B	2.85	1.6E-08
**CDP47670.1**	NAD-dependent malic enzyme (NAD-ME) (Malate dehydrogenase)	2.83	7.8E-10
**CDP48479.1**	Aldo/keto reductase	2.71	1.6E-10
**CDP48801.1**	ATP synthase gamma chain (ATP synthase F1 sector gamma subunit)	2.66	5.5E-05
**CDP48744.1**	Anaerobic glycerol-3-phosphate dehydrogenase subunit A	2.52	5.7E-10
**CDP48000.1**	NADH-quinone oxidoreductase subunit H (NADH dehydrogenase I subunit H)	2.46	1.7E-09
**CDP48900.1**	Citrate synthase	2.37	4.3E-10
**CDP48006.1**	NADH-quinone oxidoreductase chain B	2.36	6.3E-11
**CDP47836.1**	Putative isocitrate/isopropylmalate dehydrogenase	2.31	1.5E-11
**CDP47593.1**	Succinate dehydrogenase flavoprotein subunit	2.11	5.2E-12
**CDP48002.1**	NADH-quinone oxidoreductase chain F (NADH dehydrogenase I, chain F) (NDH-1, chain F)	1.99	7.1E-12
**CDP49671.1**	Putative aldo/keto reductase (oxidoreductase)	1.89	7.9E-09
**CDP49268.1**	Glycerol kinase (ATP:glycerol 3-phosphotransferase) (Glycerokinase) (GK)	1.84	2.1E-06
**CDP48742.1**	Anaerobic glycerol-3-phosphate dehydrogenase subunit C	1.83	1.1E-03
**CDP48001.1**	NADH-quinone oxidoreductase chain G (NADH dehydrogenase I, chain G)	1.77	2.6E-09
**CDP49441.1**	Glyoxylate reductase	1.77	1.9E-09
**CDP48005.1**	NADH-quinone oxidoreductase chain C (NADH dehydrogenase I, chain C)	1.76	2.8E-09
**CDP48428.1**	Pyruvate dehydrogenase E1 component (2-oxo-acid dehydrogenase E1 subunit, homodimeric type)	1.73	7.9E-12
**CDP47592.1**	Succinate dehydrogenase	1.72	3.2E-07
**CDP48009.1**	Electron transfer oxidoreductase	1.72	2.1E-02
**CDP47817.1**	Zinc-binding dehydrogenase	1.70	1.0E-03
**CDP47853.1**	NADPH:quinone reductase related Zn-dependent oxidoreductase	1.65	4.9E-04
**CDP48708.1**	Aconitase, Aconitate hydratase	1.60	8.3E-11
**CDP49161.1**	Methylmalonyl-CoA carboxytransferase 5S subunit (transcarboxylase 5S) 505 bp	1.59	1.5E-08
**CDP49666.1**	Coenzyme A transferase (Putative succinyl-CoA or butyryl-CoA:coenzyme A transferase)	1.58	8.1E-07
**CDP49604.1**	2,5-diketo-D-gluconate reductase A	1.56	5.1E-08
**CDP49580.1**	Electron transfer flavoprotein (FixA protein)	1.53	1.5E-06
**CDP48091.1**	2-oxoisovalerate dehydrogenase subunit alpha (Branched-chain alpha-keto acid dehydrogenase E1 component alpha chain) (BCKDH E1-alpha)	0.64	6.2E-03
**CDP48544.1**	Phosphate acetyltransferase	0.54	1.3E-09
**CDP49307.1**	Nitrogen-fixing NifU-like	0.52	7.0E-06
**CDP48804.1**	ATP synthase B chain (F0F1 ATP synthase subunit B)	0.45	4.8E-03
**CDP48090.1**	2-oxoisovalerate dehydrogenase subunit beta (Branched-chain alpha-keto acid dehydrogenase E1 component beta chain) (BCKDH E1-beta) Pyruvate dehydrogenase E1 component subunit beta	0.40	1.6E-10
**CDP47988.1**	Pyruvate flavodoxin/ferredoxin oxidoreductase	0.39	4.8E-06

*^(a)^Determined using a database composed of the proteome of *P. freudenreichii* CIRM-BIA129 (downloaded from NCBI.nlm.nih.gov on 23 August 2018). ^(b)^Ratio between the protein level in soy ultrafiltrate and in cow’s milk ultrafiltrate. This ratio indicates induction (ratio > 1.5) or repression (ratio < 0.66) during growth in soy ultrafiltrate.*

**TABLE 4:** Proteins in the E category (Amino acid transport and metabolism) modulated during *P. freudenreichii* growth in soy ultrafiltrate compared to cow’s milk ultrafiltrate.

Accession	Description^(a)^	Ratio^(b)^	Adjusted *p*-value
**CDP48506.1**	Alanine dehydrogenase	14.38	3.6E-17
**CDP48507.1**	D-serine/D-alanine/glycine transporter	8.85	2.1E-11
**CDP49406.1**	Glycine cleavage system T protein, aminomethyltransferase	2.95	9.9E-15
**CDP47584.1**	Tryptophan synthase beta subunit	2.81	1.4E-08
**CDP47878.1**	ATP-binding protein opuCA of Glycine betaine/carnitine/choline ABC transporter	2.45	8.3E-11
**CDP49183.1**	iolD (Myo-inositol catabolism iolD protein) (acetolactate synthase protein) [pyruvate:pyruvate acetaldehydetransferase (decarboxylating)]	2.19	1.1E-07
**CDP47555.1**	Argininosuccinate lyase (Arginosuccinase)	2.18	6.5E-04
**CDP49472.1**	ABC-type choline/glycine betaine transport, ATP-binding protein	2.14	4.1E-04
**CDP49746.1**	Delta 1-pyrroline-5-carboxylate reductase	2.11	3.1E-03
**CDP49146.1**	L-asparaginase I	1.93	2.0E-07
**CDP48778.1**	Branched-chain amino acid aminotransferase	0.66	4.3E-03
**CDP48362.1**	Monophosphatase	0.66	9.5E-06
**CDP49745.1**	Aspartate-semialdehyde dehydrogenase (Semialdehyde dehydrogenase)	0.64	1.5E-07
**CDP48347.1**	Diaminopimelate decarboxylase (DAP decarboxylase)	0.58	1.1E-05
**CDP48645.1**	Indole-3-glycerol phosphate synthase (TrpC)	0.54	2.2E-08
**CDP49386.1**	Phospho-2-dehydro-3-deoxyheptonate aldolase	0.50	7.2E-10
**CDP49437.1**	2,3,4,5-tetrahydropyridine-2,6-dicarboxylate *N*-succinyltransferase	0.49	1.4E-06
**CDP48372.1**	Binding protein of oligopeptide ABC transporter (OPN : undef : Oligopeptides)	0.46	1.5E-05
**CDP49173.1**	Dihydroxy-acid dehydratase	0.44	2.4E-10
**CDP48366.1**	D-3-phosphoglycerate dehydrogenase/erythronate 4-phosphate dehydrogenase	0.43	9.1E-08
**CDP47871.1**	Chorismate mutase	0.43	2.5E-03
**CDP48150.1**	Threonine dehydratase	0.42	8.0E-04
**CDP48503.1**	Amidohydrolase (Peptidase M20D) (Putative metal-dependent amidase/aminoacylase/carboxypeptidase)	0.42	1.7E-07
**CDP47573.1**	Phosphoribosyl-ATP pyrophosphohydrolase	0.42	1.8E-02
**CDP48773.1**	3-isopropylmalate dehydratase large subunit (Isopropylmalate isomerase) (Alpha-IPM isomerase) (IPMI)	0.38	9.1E-06
**CDP49606.1**	Shikimate 5-dehydrogenase	0.38	1.4E-08
**CDP48647.1**	Phosphoribosyl-AMP cyclohydrolase	0.37	9.2E-12
**CDP49001.1**	Glutamate dehydrogenase [NAD(P)-glutamate dehydrogenase]	0.37	2.1E-06
**CDP48467.1**	Propanediol utilization protein PduJ	0.35	7.2E-06
**CDP48419.1**	4-aminobutyrate aminotransferase	0.29	2.9E-14
**CDP49308.1**	Cysteine desulphurases, SufS	0.22	1.0E-15
**CDP49551.1**	DadA, Glycine/D-amino acid oxidases	0.22	3.6E-08
**CDP49535.1**	Cysteine synthase 2	0.19	7.2E-14
**CDP48772.1**	3-isopropylmalate dehydratase small subunit (Isopropylmalate isomerase) (Alpha-IPM isomerase) (IPMI)	0.19	2.2E-10
**CDP48346.1**	Homoserine dehydrogenase	0.17	3.5E-10

*^(a)^Determined using a database composed of the proteome of *P. freudenreichii* CIRM-BIA129 (downloaded from NCBI.nlm.nih.gov on 23 August 2018). ^(b)^Ratio between the protein level in soy ultrafiltrate and in cow’s milk ultrafiltrate. This ratio indicates induction (ratio > 1.5) or repression (ratio < 0.66) during growth in soy ultrafiltrate.*

## Discussion

Prior to using propionibacteria as probiotics in new plant-based food matrices, it was necessary to ascertain the suitability of these food matrices as substrates for growth and acidification. During this study, we focused on the behavior of the probiotic strain *Propionibacterium freudenreichii* CIRM-BIA129 in soymilk and compared it with the well-known cow’s milk medium.

In many fermented dairy products, *P. freudenreichii* grows in the presence of other bacterial species, namely LAB. Collaboration between LAB and propionibacteria is thus well documented in the context of Emmental cheese ripening. Thermophilic LAB develop in the curd during the early, and warmest, stages of cheese making. In so doing, they convert part of the dairy lactose into lactic acid. Being equipped with a known proteolytic arsenal (which propionibacteria do not have) they also convert part of the dairy proteins into peptides and amino acids, i.e., non-protein nitrogen (NPN) (Gagnaire et al., 1998, 2001a; Thierry et al., 1998). During the present work, the presence of the mesophilic *Lactobacillus plantarum* was also shown to facilitate the growth of *P. freudenreichii*, whether the growth medium was cow’s milk or soymilk (Figure 1). *P. freudenreichii* failed to grow alone in soymilk, while *L. plantarum* did. One could hypothesize that *P. freudenreichii* did not find in soymilk an appropriate source of carbon, or nitrogen, or both. However, propionibacteria are well known to ferment numerous carbohydrates. *P. freudenreichii* CIRM BIA129, which was used in this study, utilizes the lactose found in milk, as well as the glucose, galactose, D-fructose, inositol, and gluconate (Loux et al., 2015) present in plant products, but not the sucrose and raffinose that predominate in soy. By contrast, they do not display proteolytic activity (Gagnaire et al., 2001b), while some strains of *L. plantarum* do (Khalid and Marth, 1990; Duan et al., 2019). Another hypothesis could therefore be that the LAB might have degraded soy proteins into NPN, thus enabling the growth of *P. freudenreichii*. This hypothesis was confirmed using ultrafiltrates as growth media. *P. freudenreichii* grew in neither of these media. However, the addition of casein peptone to MUF, or of soy peptone to SUF, enabled propionibacteria to grow and to acidify the medium. Taken together, these results evidenced metabolic cooperation between lactobacilli and propionibacteria in soymilk, as had previously been reported in cheese (Baer, 1995; Gagnaire et al., 2001b). Selection of the propionic and LAB strains will determine this cooperation, as well as the sensory, structural and nutritional properties of the soy fermented product (Jeewanthi and Paik, 2018; Zheng et al., 2020). Concerning probiotic effects, it should be noticed that the effect of propionibacteria was often described in synergy with LAB. Indeed, mixtures of propionibacteria with lactobacilli were shown to restore normal microbiota composition and function in antibiotic-treated and in cesarean-born infants (Korpela et al., 2018). Furthermore, a perinatal use of the same mixture prevented allergic disease in a cesarean-delivered children (Kallio et al., 2019).

This research work indicated that appropriate carbohydrates were found in both media, but to a lesser extent in soy than in cow’s milk, which may explain the lower acidification observed in SUF than in MUF where lactose is not limiting. Slow and limited acidification in SUF during the growth of propionibacteria (Figure 2B) may trigger an adaptive response such as acid habituation, in line with the observed overexpression of general stress adaptation proteins and enhanced stress tolerance (see below). By contrast, the rapid and important acidification of MUF may trigger a more acute acid stress, with a different pattern of stress protein induction, and decreased stress tolerance. Slow acidification was indeed shown to trigger acid-tolerance response, which in turn allows enhanced survival of bacteria within the digestive tract (Guan and Liu, 2020; Xu et al., 2020).

**Stress tolerance was indeed clearly modulated** by the growth medium (Figure 4). Tolerance toward acid and bile salts, evaluated *in vitro*, is a key determinant of the survival of probiotics and their *in vivo* activity in propionibacteria (Lan et al., 2007). We had already shown that such tolerance could be enhanced by growing *P. freudenreichii* in hyperosmotic dairy substrates, different from laboratory media (Gagnaire et al., 2015; Huang et al., 2016). The determination of new culture conditions that further enhance tolerance to digestive stresses such as acid and bile salts offers new perspectives for the development of live and active propionibacteria probiotic fermented products. From a mechanistic point of view, this enhanced stress tolerance may be linked to an improved expression of proteins involved in defense mechanisms (V category, including ABC transporters). Indeed, growth in a soy medium enhanced the expression of proteins previously shown (Leverrier et al., 2003, 2004) to participate in the acquisition of tolerance to bile salt stress [including superoxide dismutase Mn/Fe, catalase, starvation-inducible DNA-binding protein, and thioredoxin peroxidase protein C22 (Supplementary Table S1)], in line with enhanced bile salt tolerance. Acid tolerance was enhanced by growth in soy, in line with the upregulation of typical acid stress proteins, the two subunits of methylmalonyl-CoA mutase (transcarboxylase), an enzyme which plays a central role in propionic fermentation (Wood and Werkman cycle) and acid tolerance (Leverrier et al., 2003). Other proteins previously identified as acid stress proteins (Leverrier et al., 2004), including pyruvate-flavodoxin oxidoreductase and malate dehydrogenase, were also upregulated in soy ultrafiltrate. Surprisingly, tolerance to a hydrogen peroxide challenge was drastically reduced in soy, while heat tolerance was slightly reduced. In line with this, it should be noted that the typical heat stress proteins Grol1, Grol2, DnaJ2, GrpE and Hspr2 were more strongly expressed in milk than in soy (Category O, Table 5). The RecA protein, known to participate in responses to heat and oxidative stress, was also more strongly expressed in the dairy medium, in accordance with enhanced heat and oxidative tolerance. Recent studies have thus shown that the growth medium and growth parameters (osmotic pressure, temperature, nitrogen sources, and carbohydrates) exert a major impact on the morphology, intracellular compatible solutes, stress protein expression and stress tolerance of *P. freudenreichii* (Huang et al., 2016, 2018; Gaucher et al., 2019a,c,^2020^). This has then enabled improvements to the industrial production of dried propionibacteria, in terms of their survival and stability (Huang et al., 2017; Gaucher et al., 2019b).

**TABLE 5:** Proteins in the O category (Protein turnover and chaperone) modulated during *P. freudenreichii* growth in soy ultrafiltrate compared to cow’s milk ultrafiltrate.

Accession	Description^(a)^	Ratio^(b)^	Adjusted *p*-value
**CDP48588.1**	SppA. Periplasmic serine proteases	6.05	5.7E-09
**CDP47990.1**	Metalloprotease (Peptidase family M13)	2.86	2.5E-03
**CDP47983.1**	Thioredoxin peroxidase/Alkyl hydroperoxide reductase protein C22/General stress protein 22	2.29	1.2E-13
**CDP49595.1**	FK506-binding protein (peptidyl-prolyl cis-trans isomerase)	2.07	1.5E-07
**CDP49391.1**	Probable peptidyl-prolyl *cis-trans* isomerase A	1.81	3.5E-10
**CDP48377.1**	Zn dependant peptidase	1.62	3.0E-04
**CDP49065.1**	Surface layer protein A (S-layer protein A)	1.56	3.1E-07
**CDP47874.1**	60 kDa chaperonin 1 (Protein Cpn60 1) (groEL protein 1) (Heat shock protein 60 1)	0.66	2.2E-07
**CDP49125.1**	60 kDa chaperonin 2 (Protein Cpn60 2) (groEL protein 2) (Heat shock protein 60 2)	0.65	1.6E-07
**CDP48880.1**	Thioredoxin reductase	0.64	1.7E-05
**CDP48879.1**	Thioredoxin	0.62	6.1E-04
**CDP48050.1**	Chaperone protein dnaJ 2 (DnaJ2 protein) (Heat shock protein 40 2)	0.61	6.4E-06
**CDP48051.1**	Protein GrpE 2 (HSP-70 cofactor 2) (Co-chaperone protein GrpE2)	0.58	6.8E-04
**CDP48878.1**	OsmC protein	0.53	1.0E-07
**CDP49021.1**	Protein GrpE 1 (HSP-70 cofactor 1) (Co-chaperone protein GrpE1)	0.49	7.6E-05
**CDP49400.1**	HesB protein	0.38	1.9E-12
**CDP47885.1**	Putative *O*-sialoglycoprotein endopeptidase	0.37	4.8E-08
**CDP49311.1**	FeS assembly protein SufD	0.36	3.6E-13
**CDP49309.1**	ABC-type transport system involved in Fe–S cluster assembly. ATPase component. SufC	0.33	4.2E-14
**CDP49312.1**	FeS assembly protein SufB	0.29	2.2E-13
**CDP48273.1**	Surface layer protein B	0.17	7.1E-12
**CDP48858.1**	Surface layer protein E	0.16	4.1E-12

*^(a)^Determined using a database composed of the proteome of *P. freudenreichii* CIRM-BIA129 (downloaded from NCBI.nlm.nih.gov on 23 August 2018). ^(b)^Ratio between the protein level in soy ultrafiltrate and in cow’s milk ultrafiltrate. This ratio indicates induction (ratio > 1.5) or repression (ratio < 0.66) during growth in soy ultrafiltrate.*

**The morphology of *P. freudenreichii* was markedly affected** by the growth medium in terms of size, shape and extracellular matrix. Bacterial shape is subject to changes that may be either cyclic (division cycles) or sporadic in response to changing conditions, including the chemical environment, physical constraints, nutrient availability and other environmental parameters (van Teeseling et al., 2017). Indeed, starvation may favor a shift from a rod to a coccoid shape, while stress adaptation may lead to an increase in cell length. During our study, nutrient availability differed drastically depending on the growth medium. When cultivated in soy, propionibacteria displayed reduced thickness after drying on a mica surface, as shown by AFM (Figure 3). This significant deformation of the soy-grown propionibacteria following dehydration was indicative of major changes to the composition and/or permeability of the cell wall, and reduced mechanical resistance to constraint. This was consistent with the observed upregulation, in soy, of several proteins involved in envelope biogenesis (M category, Figure 6), including UDP-*N*-acetylmuramate-L-alanine ligase, MurC, an essential, cytoplasmic peptidoglycan biosynthetic enzyme, and UDP-galactopyranose mutase, also involved in cell wall construction. *P. freudenreichii* also appeared to be embedded in an extracellular matrix when grown in MUF. The nature of this matrix is still unknown. However, it is possible to speculate that this may correspond to exopolysaccharides. Indeed, an EPS capsule was described in *P. freudenreichii* (Deutsch et al., 2010, 2012) and its biosynthesis was shown to be modulated by environmental factors. Moreover, EPS production by propionibacteria has already been shown to be enhanced in cow’s milk permeate when compared to laboratory media (Gorret et al., 2001a,b). Accordingly, the availability of lactose, which is abundant in MUF, was shown to boost production of EPS in other bacteria (Bauer et al., 2009; Sardari et al., 2017; Cirrincione et al., 2018). Adaptation to acid and salt has also been shown to increase EPS production in *P. acidipropionici* (Cavero-Olguin et al., 2019), as has the exposure to moderate acid pH (5.0) of *Lactobacillus helveticus* (Torino et al., 2001). Moreover, the marked impact of the growth medium on the biosynthesis of extractable surface layer proteins (SlpA, SlpB, SlpE, LspA), which was here enhanced in the cow’s milk medium, may also play a role in morphological changes affecting *P. freudenreichii.* Variations in extracellular compounds such as EPS and surface layer proteins may also modulate stress tolerance (Sleytr et al., 2014; Caggianiello et al., 2016).

**Changes to the surface proteome suggest a modulation of probiotic properties**. Surface extractable proteins of the S-layer type were shown here to be more abundant in MUF than in SUF cultures. This may have been triggered by different degrees of acid stress in these two media (see above). Indeed, the synthesis of S-layer proteins has previously been reported to respond to environmental stress conditions in *Lactobacillus acidophilus* (Grosu-Tudor et al., 2016; Palomino et al., 2016), with increased expression following moderate doses of stress but decreased expression after acute doses of stress (Khaleghi et al., 2010). We had previously shown that extractable surface proteins play a key role in interactions between *P. freudenreichii* and the host (do Carmo et al., 2017, 2019), as is the case in many probiotic bacteria (do Carmo et al., 2018a). The extraction of such proteins suppresses *P. freudenreichii* immunomodulation (Le Marechal et al., 2015). The mutation of *slpB*, encoding one of these surface proteins, suppresses immunomodulation (Deutsch et al., 2017) as well as adhesion to host cells (do Carmo et al., 2017), and has many pleiotropic effects on the surface properties of *P. freudenreichii* (do Carmo et al., 2018b). In the present study, the expression of *P. freudenreichii* surface extractable proteins (Figure 5 and Table 1), including SlpB and SlpA, suggested that interactions with host cells might be modified, with reduced adhesion to host cells (do Carmo et al., 2017) and unpredictable immunomodulatory properties. Indeed, in the well-known probiotic *Lactobacillus acidophilus*, a shift in the expression of surface-layer proteins was seen to drastically modify its immunomodulatory properties (Konstantinov et al., 2008). In *P. freudenreichii*, the effects of the down-regulation of Slp proteins following growth in a soy medium on its probiotic abilities (stress tolerance, persistence, immunomodulation) therefore need to be investigated *in vivo*.

**Adaptation to the different media and the substrates that they provide** was illustrated by the whole-cell proteomic analysis (Figure 6). Saccharides differ in terms of their composition and abundance in soymilk (sucrose, stachyose, raffinose, glucose, fructose, verbascose, arabinose, rhamnose) and cow’s milk (lactose). This may determine different metabolic adaptations that can be evidenced using proteomics. Concerning carbohydrates transport and metabolism (G Category), major differences were indeed observed (Figure 6 and Table 2), with 26 upregulated proteins in the soy medium, in line with the greater variety of sugars it contained, when compared to cow’s milk. This included 6 proteins involved in glycolysis/gluconeogenesis (phosphoglycerate mutase; phosphoglucose isomerase; phosphoglucomutase, glucose-6-phosphate isomerase; phosphoglycerate mutase and polyphosphate glucokinase). Furthermore, 9 proteins of the pentose phosphate pathway were also enhanced (glucose-6-phosphate deshydrogenase; phosphoketolase pyrophosphate; ribose-5-phosphate isomerase; transketolase; ribose-5-phosphate isomerase; gluconate kinase; ribulose-5-phosphate 3-epimerase, transaldolase). This suggested the utilization of soy sugars via the pentose phosphate pathway, supplying pentose for nucleotide metabolism. Soy-upregulated proteins included glycosyltransferase, which is involved in glycogen synthesis. They also included 3 enzymes (IolC; IolD; IolG) involved in the utilization of myo-inositol, which is present in soybean and a constituent of phytates. Interestingly, an ability to metabolize inositol may facilitate the digestibility of soymilk thanks to propionibacterial fermentation and improve mineral availability by decreasing the mineral chelating capacity of the phytate. By contrast, only 6 proteins of the carbohydrate transport and metabolism “G” category, were enhanced in the dairy medium, including a glycogen debranching enzyme, 2 proteins involved in glycolysis (triosephosphate isomerase; phosphoglycerate mutase) and 3 involved in galactose metabolism (α-glucosidase; NAD-dependent epimerase; nucleoside-diphosphate-sugar epimerase). A similar approach recently evidenced adaptation of the water kefir-borne *L. hordei* to sucrose, including the modulation of proteins involved in carbohydrate metabolism, providing fundamental knowledge for its use as a starter culture in plant-based food fermentations with *in situ* dextran formation (Bechtner et al., 2020).

In accordance with the G Category proteins found, growth in a soy environment resulted in the upregulation of 38 proteins in the “energy production and conversion” C Category (Figure 6 and Table 3). They are involved in glycolysis, Krebs (TCA) cycle, glutamate metabolism, pentose metabolism, electron transfer and oxidative phosphorylation, glyoxylate and dicarboxylate metabolism, propionic fermentation (propanoate metabolism), glycerol degradation and glycerophospholipid metabolism. Growth in the dairy medium resulted in the upregulation of 6 proteins in the “energy production and conversion” C Category, involved in valine, leucine and isoleucine degradation and in pyruvate metabolism (Figure 6 and Table 3). Concerning amino acid transport and metabolism (E Category and Table 4), 25 proteins were more markedly expressed in the dairy medium. These proteins were related to the metabolism of amino acids such as leucine, isoleucine, valine, glycine, serine and threonine. In particular, members of the shikimate pathway, responsible for the biosynthesis of phenylalanine, tyrosine and tryptophan, were enhanced. By contrast, growth in the soy medium resulted in the upregulation of only 10 proteins, involved in the active transport of amino acids such as Ser, Ala, Gly or derived compounds such as glycine betaine, carnithine and choline. Proteins involved in the catabolism of alanine, Gly, Ser, arginosuccinate, proline and asparagine were also enhanced in soy. This is in line with a higher content of Ser, Ala and Gly in soy proteins, and with a higher content of Val and Ile in dairy proteins (Gorissen et al., 2018), and may reflect the metabolic adaptation of propionibacteria to the amino acids available.

## Conclusion

This work constitutes, to the authors’ knowledge, the first report of the development of a soymilk fermented by propionibacteria. It indicates that it is possible to produce various functional fermented soy products using probiotic dairy propionibacteria. Several propionibacteria-fermented dairy products, including cheeses and fermented cow’s milk or whey were previously described. Indeed, *P. freudenreichii* can be used to generate fermented milks without any adverse effects on the product characteristics in terms of sensory properties (Yerlikaya et al., 2020). *P. freudenreichii*-fermented milks may constitute a source of riboflavin (LeBlanc et al., 2006), synergize drug treatments (Cousin et al., 2016), or counteract the deleterious effects thereof (Cordeiro et al., 2018; do Carmo et al., 2019). However, consumers are now looking for non-dairy functional foods, and fermented soymilk offers an alternative source of live probiotics. This work thus opens avenues for new food products and supplements, which will be adequate for vegans, flexitarians and reducetarians, while keeping the benefits of propionic fermentation.

This work also evidenced the collaboration between two food-grade bacteria, namely *P. freudenreichii* and *L. plantarum*, in a soy medium. This is also new, although these two bacterial species were already shown to grow together in corn silage (Abdul Rahman et al., 2017). *P. freudenreichii* CIRM-BIA129 does not grow in soymilk, and nor do several other tested propionibacteria strains (data not shown). *Lactobacillus plantarum* CIRM-BIA465 grows in soymilk, as do several other tested strains (data not shown). Other lactobacilli, including *L. pentosus, L. plantarum, L. rhamnosus, L. amylovorus, L. coryniformis, L. kunkeei*, and *L. curvatus*, were recently shown to ferment soymilk as a substrate (Harlé et al., 2020). Co-culture with an appropriate lactic acid bacterium will clearly facilitate propionibacterial growth thanks to metabolic complementarities. This opens avenues for the development of innovative fermented plant-based products with symbiotic lactobacilli and propionibacteria in non-dairy analogs. This will also allow combining probiotic effects of both lactobacilli and propionibacteria. Indeed, whey fermented by *P. freudenreichii* was shown to alleviate symptoms of ulcerative colitis in humans (Suzuki et al., 2006). *L. plantarum* 299v, either in a capsule (Ducrotté et al., 2012), or in a fruit juice (Nobaek et al., 2000), alleviates symptoms of irritable bowel syndrome, and this is recognized with “strong evidence of effect” on this ailment (Sniffen et al., 2018). Combining *P. freudenreichii* and *L. plantarum* opens new perspectives for the development of fermented products with synergistic effects. As a precedent, a probiotic food supplement combining two anti-inflammatory bacteria, including one *L. plantarum* strain, modulated peripheral immune response in children with celiac disease autoimmunity, suggesting a limitation of the progression of disease development, as a result of synergistic immunomodulatory effects (Håkansson et al., 2019). Another complex probiotic product containing *P. freudenreichii* in conjunction with *Lactobacillus rhamnosus* and *Bifidobacterium breve* protected cesarean-delivered children from allergic disease and eczema (Kallio et al., 2019).

Shifting from dairy to a soy environment exerted pleiotropic effects on propionibacteria. This included modulation of the proteome toward overexpression of proteins involved in transport and metabolism of soy substrates, as well as modulation of stress adaptation proteins. Accordingly, digestive stress tolerance was enhanced as a result of growth in soy medium. This medium also triggered rearrangements of key surface compounds known to play a determinant role in probiotic/host interactions. This may in turn affect the probiotic properties of dairy propionibacteria. This now needs to be investigated *in vitro* and *in vivo* for the development of new fermented functional foods.

## Data Availability Statement

The data can be found here: https://doi.org/10.15454/G6NGSZ.

## Author Contributions

FT, FGa, FGu, JJ, and VB-B performed the experiments. GJ and VG designed the experiments and supervised the work. FC and NI processed the data and designed the figures and tables. All the authors participated in writing the manuscript.

## Conflict of Interest

FGa was employed by the French company Bioprox. The remaining authors declare that the research was conducted in the absence of any commercial or financial relationships that could be construed as a potential conflict of interest.

## Supplementary Material

Supplementary Table S1 Complete list of proteins identified during this study of *P. freudenreichii* growth in soy ultrafiltrate and cow’s milk ultrafiltrate. Ratios were calculated using the eXtracted ion Current method.
